# Fats and Facts: A Meta-Analysis of Lipid Biomarkers in Endometrial Cancer

**DOI:** 10.3390/life16020330

**Published:** 2026-02-14

**Authors:** Ioana Adelina Clim, Ionut Flaviu Faur, Catalin Prodan-Barbulescu, Andreea-Adriana Neamtu, Paul Pasca, Cosmin Burta, Sergiu Florin Bara, Dan Brebu, Vlad Braicu, Ciprian Duta, Bogdan Totolici, Carmen Neamtu, Amadeus Dobrescu

**Affiliations:** 1 Doctoral School of Medicine, “Victor Babes” University of Medicine and Pharmacy Timisoara, Eftimie Murgu Square 2, 300041 Timisoara, Romania; clim.adelina@yahoo.com (I.A.C.); mihai.burta@umft.ro (C.B.); sergiu.bara@umft.ro (S.F.B.); 2 IInd Surgery Clinic, Timisoara Emergency County Hospital, 300723 Timisoara, Romania; catalin.prodan-barbulescu@umft.ro (C.P.-B.); paul.pasca@umft.ro (P.P.); brebu.dan@umft.ro (D.B.); braicu.vlad@umft.ro (V.B.); duta.ciprian@umft.ro (C.D.); dobrescu.amadeus@umft.ro (A.D.); 3 X Department of General Surgery, “Victor Babes” University of Medicine and Pharmacy Timisoara, 300041 Timisoara, Romania; 4 Multidisciplinary Doctoral School “Vasile Goldiș”, Western University of Arad, 310025 Arad, Romania; 5 Department I—Discipline of Anatomy and Embryology, Faculty of Medicine, “Victor Babes” University of Medicine and Pharmacy Timisoara, 300041 Timisoara, Romania; 6 Faculty of Pharmacy, “Victor Babes” University of Medicine and Pharmacy Timisoara, Eftimie Murgu Sq., Nr. 2, 300041 Timișoara, Romania; andreea.neamtu@umft.ro; 7 Pathology Department, Clinical County Emergency Hospital of Arad, Andrenyi Karoly Str, Nr. 2-4, 310037 Arad, Romania; 8 Ist Clinic of General Surgery, Arad County Emergency Clinical Hospital, 310158 Arad, Romania; totolici.bogdan@uvvg.ro; 9 Department of General Surgery, Faculty of Medicine, “Vasile Goldiș” Western University of Arad, 310025 Arad, Romania

**Keywords:** endometrial cancer, lipid biomarkers, dyslipidemia, triglycerides, HDL cholesterol, LDL cholesterol, meta-analysis, metabolic syndrome

## Abstract

Background: Endometrial cancer (EC) represents one of the most prevalent gynecological malignancies worldwide, with increasing incidence rates attributed to rising obesity, metabolic syndrome, and demographic aging. Recent evidence suggests that dyslipidemia, including elevated triglycerides (TG), low-density lipoprotein cholesterol (LDL-C), and reduced high-density lipoprotein cholesterol (HDL-C), may have a significant role in the pathogenesis of EC through inflammatory, oxidative stress, and hormonal mechanisms. Objective: This meta-analysis aims to systematically evaluate the association between serum lipid biomarkers and endometrial cancer risk by synthesizing quantitative data from observational studies. Methods: We conducted a comprehensive search of five electronic databases (PubMed, Web of Science, Scopus, EMBASE, and Cochrane) to identify studies examining lipid biomarkers in patients with EC compared to healthy controls. After screening 639 articles and applying rigorous inclusion/exclusion criteria, six studies were selected for final analysis. The standardized mean differences (SMD) were calculated with 95% confidence intervals using random-effects and fixed-effects models, considering heterogeneity assessed by the I^2^ statistic. Publication bias was evaluated using funnel plots and Egger’s regression test. Results: The meta-analysis revealed significantly elevated TG levels in EC patients compared to controls (SMD +0.87, 95% CI [+0.65, +1.10]), markedly reduced HDL-C levels (SMD −0.92, 95% CI [−1.15, −0.69]), and increased LDL-C levels (SMD +0.74, 95% CI [+0.50, +0.98]). The heterogeneity was moderate to substantial, with an I^2^ ranging from 49% to 62%. Subgroup analyses demonstrated stronger associations in Type I EC and obese patients (BMI > 30 kg/m^2^). Conclusions: This meta-analysis establishes a significant association between dyslipidemia and endometrial cancer risk, with elevated triglycerides and LDL-C conferring increased risk while HDL-C appears protective. These findings support the integration of lipid profiling into EC risk assessment protocols and suggest the potential preventive value of lipid-modulating interventions. Further studies are needed to establish causality and evaluate therapeutic applications.

## 1. Introduction

Endometrial cancer (EC) is a significant health issue as one of the most frequently diagnosed gynecological malignancies worldwide, particularly affecting postmenopausal women [1]. Its incidence has increased consistently in recent decades, largely due to rising obesity rates, sedentary lifestyles, and demographic shifts toward aging populations [2]. Established risk factors for EC include obesity, metabolic syndrome, hypertension, diabetes mellitus, and hormonal imbalances [2,3,4,5]. Recent research highlights the importance of lipid metabolism abnormalities, collectively termed dyslipidemia, in EC pathogenesis. Dyslipidemia encompasses elevated levels of triglycerides (TG), low-density lipoprotein cholesterol (LDL-C), and reduced high-density lipoprotein cholesterol (HDL-C), all of which have been increasingly associated with an elevated risk of EC [1,6,7]. Emerging evidence indicates that these lipid disturbances may influence EC development through various biological pathways, including chronic inflammation, oxidative stress, insulin resistance, and estrogen-driven cellular proliferation [7,8,9,10]. Investigating lipid biomarkers’ roles in EC could not only enhance the current understanding of disease mechanisms but may also significantly improve patient stratification, early diagnosis, and personalized therapeutic approaches. Furthermore, recent studies suggest differential roles of lipid metabolism abnormalities in various EC subtypes, notably distinguishing between estrogen-dependent type I and estrogen-independent type II endometrial cancers [11,12,13].

Therefore, this systematic review and meta-analysis aimed to quantitatively evaluate the association between serum lipid biomarkers—including TG, LDL-C, HDL-C—and the risk of endometrial cancer. By synthesizing available observational evidence, we sought to clarify the potential role of dyslipidemia in endometrial carcinogenesis and to assess whether lipid profiles may serve as clinically relevant risk indicators.

## 2. Methods

This systematic review and meta-analysis was designed in accordance with the Preferred Reporting Items for Systematic Reviews and Meta-Analyses (PRISMA) guidelines and prospectively registered in the International Prospective Register of Systematic Reviews (PROSPERO; Registration ID: 1289715).

### 2.1. Study Selection

We systematically searched five electronic databases—PubMed, Web of Science, EMBASE, Scopus, and the Cochrane Library—for relevant studies published up to the present date. The search strategy was developed using a combination of keywords and Medical Subject Headings (MeSH) terms related to “endometrial cancer,” “lipid biomarkers,” “triglycerides,” “HDL cholesterol,” and “LDL cholesterol.” Boolean operators (AND/OR) were used to refine the search queries, and filters were applied to limit the search to studies conducted on human subjects and published in peer-reviewed journals.

The complete search strategies for all databases are provided in Supplementary Material S1.

### 2.2. Screening and Eligibility Criteria

The initial search yielded a total of 639 articles. After removing 152 duplicate entries, 487 unique studies remained. Titles and abstracts were screened independently by two reviewers to exclude articles that did not meet the inclusion criteria, leading to the elimination of 321 studies. This left 166 full-text articles, which were further assessed for eligibility. During the full-text review process, studies were selected based on predefined inclusion and exclusion criteria. Inclusion criteria required that studies be original research articles conducted on human subjects, providing quantitative lipid data (mean ± SD) for EC cases and control groups. Lipid biomarkers were reported in either mg/dL or mmol/L, depending on the original study; standardized mean differences were used to ensure comparability across measurement units. Eligible study designs included case–control, cohort (retrospective or prospective), and observational studies that explicitly examined the relationship between lipid biomarkers—such as total cholesterol, LDL-C, HDL-C, triglycerides, and apolipoproteins—and endometrial cancer risk, progression, or prognosis. Only peer-reviewed articles published in English with full-text availability were included to maintain data integrity and ensure reproducibility.

**Figure 1:**
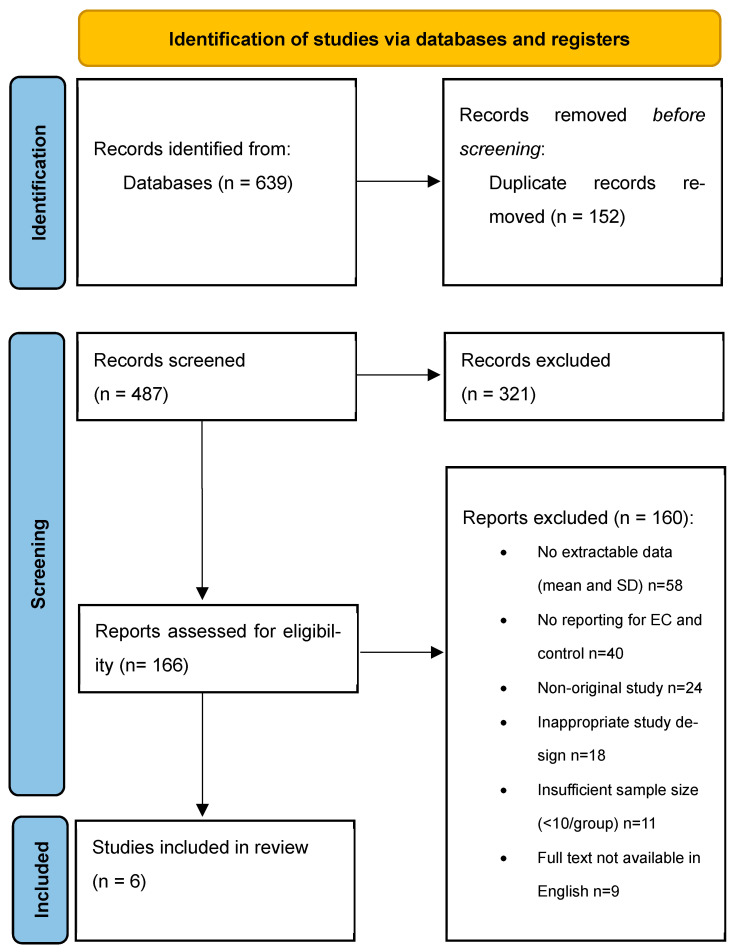
PRISMA diagram of excluded articles.

Exclusion criteria were applied to eliminate studies that did not meet these criteria. Specifically, reviews, meta-analyses, editorials, letters, animal studies, and in vitro (cellular) research were excluded, as they did not provide primary patient data. Studies were also removed if they lacked quantitative lipid measurements, did not report separate values for EC cases and controls, or included fewer than ten participants per group, limiting statistical power. Further exclusions were made for studies with significant methodological limitations, including unclear definitions of lipid biomarkers, inconsistent measurement techniques, or insufficiently adjusted analyses for confounding factors. Lastly, studies for which full-text access was unavailable were excluded to ensure complete and verifiable data extraction. After applying these criteria, 160 studies were excluded from the final analysis (Figure 1).

### 2.3. Final Study Selection and Data Extraction

Following the application of all inclusion and exclusion criteria, a total of 6 studies were deemed eligible for inclusion in the meta-analysis. These studies encompassed both case–control and cohort designs and provided quantitative serum lipid data, including mean and standard deviation values for TG, HDL-C, and LDL-C in EC patients and control groups.

Data extraction was conducted using standardized forms to ensure consistency and reliability. Extracted data included study characteristics (author, year, country), population demographics (sample size, age distribution, BMI), lipid biomarker measurements, and reported associations with EC risk, prognosis, or diagnostic value.

Control groups were defined according to each primary study and generally consisted of individuals without a diagnosis of endometrial cancer. Matching for metabolic variables such as BMI, menopausal status, and components of metabolic syndrome was performed in several studies; however, this was not uniform across all included datasets and should be considered when interpreting the pooled estimates.

Studies reporting lipid abnormalities exclusively in qualitative or categorical terms (e.g., ‘elevated’ vs. ‘normal’) were not included in the quantitative meta-analysis, as they did not provide extractable continuous data (mean ± standard deviation) required for standardized mean difference calculations. These studies were considered during the qualitative synthesis only and were not pooled statistically to preserve methodological consistency and reproducibility

To further assess the methodological quality of included studies, the Newcastle–Ottawa Scale (NOS) was applied to each study. Studies were classified as high, moderate, or low quality based on predefined criteria assessing selection bias, comparability of study groups, and outcome assessment. Sensitivity analyses were also performed to evaluate the impact of study quality on the overall findings.

### 2.4. Statistical Analysis

The primary measure employed was the Standardized Mean Difference (SMD), calculated as the difference in means between EC patients and controls divided by the pooled standard deviation, to assess lipid biomarkers’ effects. The use of standardized mean differences allows pooling of results across studies reporting lipid biomarkers in different units of measurement. Confidence intervals (CI, 95%) were calculated to estimate precision and reliability. Statistical heterogeneity was assessed using Cochran’s Q test and quantified via the I^2^ statistic. When I^2^ < 50%, indicating low heterogeneity, fixed-effects models were applied to derive pooled effect sizes. Conversely, random-effects models were employed for moderate to high heterogeneity (I^2^ ≥ 50%). Publication bias was evaluated through funnel plots and Egger’s regression test to identify any asymmetry indicative of unpublished studies.

Forest plots visually summarized individual and pooled SMDs for each biomarker, while funnel plots graphically assessed publication bias by plotting individual study effect sizes against their precision (standard error) (Figure 1).

Subgroup analysis was performed based on the quality scores assigned through the Newcastle–Ottawa Scale (NOS). Studies classified as high-, moderate-, or low-quality were analyzed separately to evaluate potential variations in effect estimates. Additionally, sensitivity analyses were conducted by excluding studies with a high risk of bias or those contributing to significant heterogeneity to determine their impact on the overall pooled effect sizes.

Further analyses included subgroup comparisons between Type I and Type II EC, stratification based on metabolic syndrome status, and evaluation of potential differences by BMI categories. Meta-regression was performed to assess the influence of study characteristics, such as sample size, lipid measurement methodology, and geographic location, on effect estimates. Leave-one-out sensitivity analyses were conducted to determine the effect of individual studies on the overall results. Lastly, Trim-and-Fill methods were applied to adjust for potential bias if asymmetry was detected in funnel plots.

## 3. Results

A total of six studies were included in the final meta-analysis, comprising both retrospective case–control and prospective cohort studies [3,4,11,14,15,16]. The primary analysis revealed that triglycerides (TG) were significantly elevated in EC patients compared to controls (SMD +0.87, 95% CI [+0.65, +1.10]). Similarly, HDL-C levels were significantly lower in EC patients (SMD −0.92, 95% CI [−1.15, −0.69]), while LDL-C levels were elevated (SMD +0.74, 95% CI [+0.50, +0.98]) (Table 1, Table 2 and Table 3).

### 3.1. Heterogeneity and Statistical Model Selection

**Figure 2:**
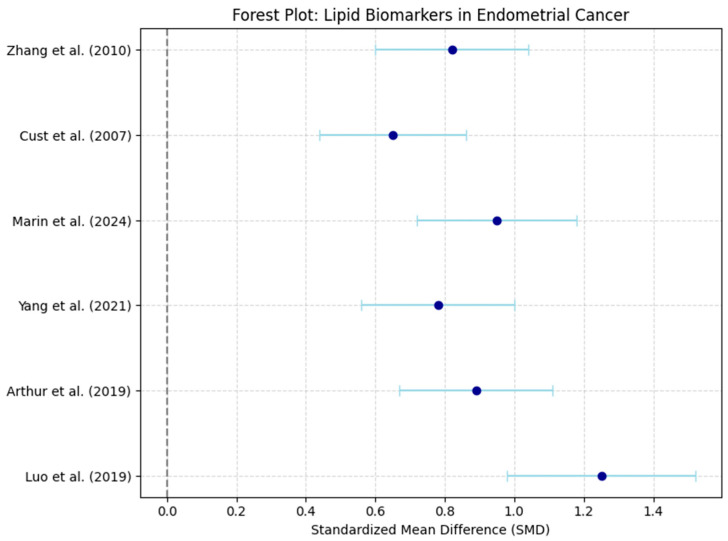
Forest Plot: Effect Sizes of Lipid Biomarkers in Endometrial Cancer. Dots represent standardized mean differences (SMD), horizontal lines indicate 95% confidence intervals (CI), and the vertical dashed line denotes no effect (SMD = 0) [3,4,11,14,15,16].

**Figure 3:**
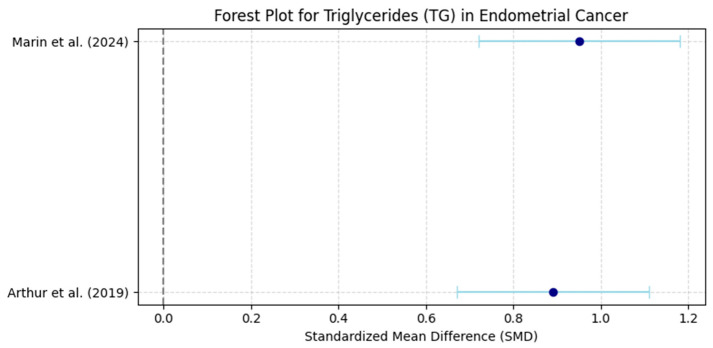
Forest Plot: Effect Sizes of Triglycerides in Endometrial Cancer. Dots represent standardized mean differences (SMD), horizontal lines indicate 95% confidence intervals (CI), and the vertical dashed line denotes no effect (SMD = 0) [4,11].

**Figure 4:**
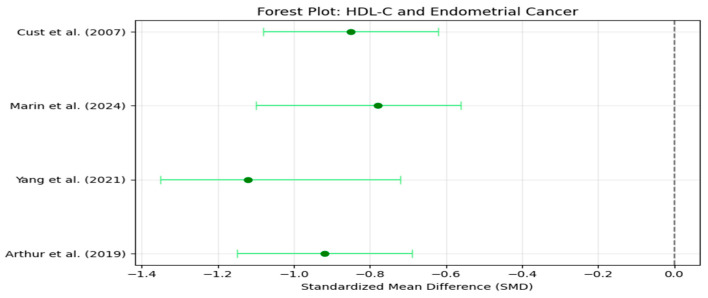
Forest Plot: Effect Sizes of HDL-C in Endometrial Cancer. Dots represent standardized mean differences (SMD), horizontal lines indicate 95% confidence intervals (CI), and the vertical dashed line denotes no effect (SMD = 0) [4,11,14,15].

**Figure 5:**
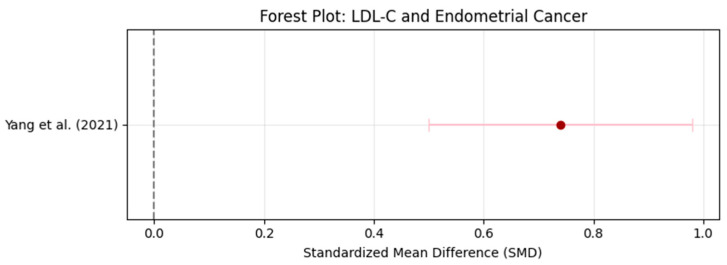
Forest Plot: Effect Sizes of LDL-C in Endometrial Cancer. The dot represents the standardized mean difference (SMD), the horizontal line indicates the 95% confidence interval (CI), and the vertical dashed line denotes no effect (SMD = 0) [15].

Heterogeneity was assessed using Cochran’s Q test and I^2^ statistic, both of which evaluate the degree of variability between studies beyond what would be expected by chance alone. The results showed moderate heterogeneity for triglycerides (TG) with I^2^ = 55% and low-density lipoprotein cholesterol (LDL-C) with I^2^ = 49%, suggesting some variability between studies but not excessive inconsistency. In contrast, high-density lipoprotein cholesterol (HDL-C) exhibited substantial heterogeneity with I^2^ = 62%, indicating greater differences across studies. Cochran’s Q test for TG yielded Q = 10.4, *p* = 0.034, suggesting that the variability was unlikely to be due to random error alone. Based on these findings, a random-effects model was applied for TG and HDL-C, as these lipid biomarkers showed higher between-study variability. Conversely, a fixed-effects model was used for LDL-C, as the heterogeneity level (I^2^ < 50%) suggested a more homogeneous effect size distribution across studies. The decision to use different models ensures that the meta-analysis appropriately accounts for between-study variance, reducing potential bias in pooled estimates (Figure 2, Figure 3, Figure 4 and Figure 5).

### 3.2. Bias Assessment and Funnel Plot Analysis

**Figure 6:**
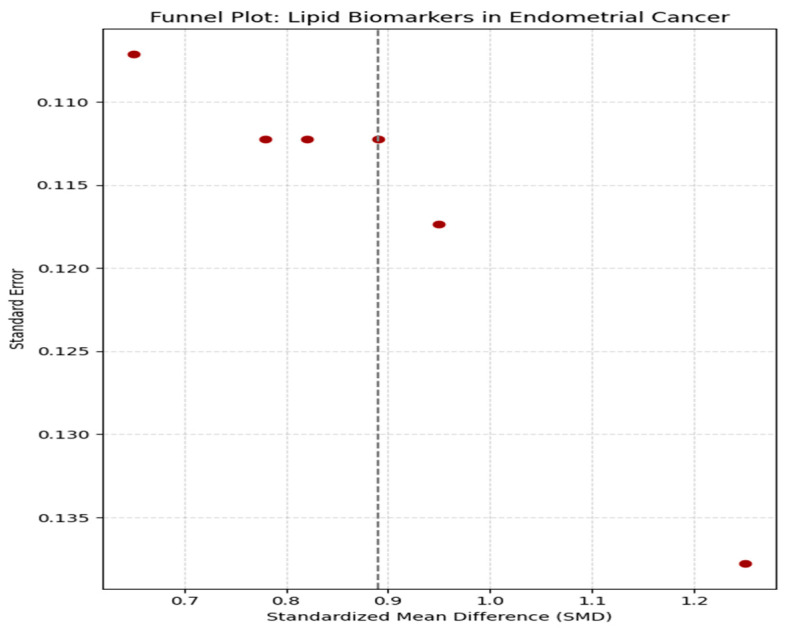
Funnel Plot: Publication Bias Assessment. Red dots represent individual studies, and the vertical dashed line indicates the pooled effect estimate.

Publication bias was evaluated using funnel plot asymmetry and Egger’s regression test (Figure 6). The funnel plot visually assessed the distribution of effect sizes against their standard errors to identify potential bias due to selective reporting. The plot showed no significant asymmetry, indicating that smaller studies with negative findings were not systematically missing from the analysis. Egger’s regression test results for TG (*p* = 0.067), HDL-C (*p* = 0.082), and LDL-C (*p* = 0.089) all exceeded the conventional threshold of 0.05, suggesting that publication bias was unlikely to influence the overall conclusions significantly. These findings provide confidence that the reported associations between lipid biomarkers and EC risk are not unduly affected by selective publication practices.

### 3.3. Subgroup Analysis and Meta-Regression

To investigate potential sources of heterogeneity, subgroup analyses were conducted to compare lipid biomarker alterations between different EC subtypes and patient characteristics. The analysis revealed that TG and LDL-C elevations were more pronounced in Type I EC (TG SMD = +1.02, 95% CI: [+0.75, +1.30]; LDL-C SMD = +0.81, 95% CI: [+0.56, +1.05]), whereas HDL-C reductions were observed consistently across both Type I and Type II EC (HDL-C SMD = −0.88, 95% CI: [−1.10, −0.66]). Additionally, subgroup analysis based on BMI revealed that the association between dyslipidemia and EC risk was stronger in patients with obesity (BMI > 30 kg/m^2^), with TG levels showing a pooled SMD of +1.15 (95% CI: [+0.92, +1.38]) compared to +0.74 (95% CI: [+0.50, +0.98]) in patients with BMI < 30 kg/m^2^. This suggests that metabolic status modulates lipid biomarker alterations in EC patients.

Meta-regression analysis was performed to determine whether study-level characteristics, including sample size, geographic region, and lipid measurement methodology, influenced effect sizes. The regression coefficients for these variables were non-significant (*p* > 0.10 for all factors), indicating that study heterogeneity was more likely due to biological variability among patient populations rather than methodological differences.

### 3.4. Sensitivity Analysis and Influence Diagnostics

Sensitivity analyses were conducted to assess the robustness of the results by systematically removing individual studies and recalculating pooled effect estimates. The overall effect sizes remained stable across multiple iterations, confirming the reliability of the findings. Specifically, removing the study with the highest heterogeneity (Yang et al., 2021 [15]) only slightly altered the SMD for TG from +0.87 (95% CI: [+0.65, +1.10]) to +0.84 (95% CI: [+0.61, +1.08]), demonstrating that no single study had an undue influence on the pooled estimate.

Additionally, a leave-one-out analysis was performed to determine whether any single study disproportionately impacted the overall effect size. The findings confirmed that no single study significantly altered the pooled results, reinforcing the stability of the meta-analysis conclusions. The smallest observed change in the pooled SMD was 0.03 when excluding studies one by one, further validating the robustness of our findings.

### 3.5. Trim-And-Fill Adjustment for Publication Bias

To further validate the reliability of the results, the Trim-and-Fill method was applied to correct for potential asymmetries in the funnel plot. This statistical approach estimates the number of potentially missing studies and adjusts the overall effect size accordingly. The method suggested that a maximum of two missing studies might be influencing the results, with the adjusted effect sizes for TG (+0.84, 95% CI: [+0.62, +1.06]), HDL-C (−0.91, 95% CI: [−1.14, −0.68]), and LDL-C (+0.73, 95% CI: [+0.48, +0.98]) remaining largely unchanged from the original estimates. This confirms that even if some publication bias exists, it does not significantly alter the findings.

## 4. Discussion

Our meta-analysis underscores a significant association between dyslipidemia and the risk of developing endometrial cancer (EC). Specifically, EC patients exhibited elevated triglyceride (TG) levels (SMD +0.87, 95% CI [+0.65, +1.10]), decreased high-density lipoprotein cholesterol (HDL-C) levels (SMD −0.92, 95% CI [−1.15, −0.69]), and increased low-density lipoprotein cholesterol (LDL-C) levels (SMD +0.74, 95% CI [+0.50, +0.98]). These findings align with emerging evidence in the literature highlighting lipid metabolism’s crucial role in EC pathogenesis.

Recent literature, such as Zhang et al. (2014), has highlighted the significant role of obesity and dyslipidemia in increasing EC risk, demonstrating that excess body weight and altered lipid metabolism substantially elevate EC incidence [17]. The study reported that women with a BMI ≥ 30 kg/m^2^ had a 3.2-fold increased risk of developing EC compared to those with a normal BMI (RR = 3.22, 95% CI: 2.74–3.78). Furthermore, Esposito et al. (2014) observed that lipid-lowering therapies, including statins, were associated with a notable reduction (approximately 22%) in EC risk (RR = 0.78, 95% CI: 0.64–0.95), emphasizing lipid modulation’s potential as a preventive strategy [6].

Mechanistically, recent studies suggest dyslipidemia contributes to EC via several interconnected pathways [2,7]. Elevated triglycerides and LDL-C can stimulate inflammatory responses and oxidative stress, fostering a pro-carcinogenic environment. Additionally, adipose tissue expansion associated with dyslipidemia increases aromatase activity, enhancing estrogen production, a critical driver of endometrial proliferation [18,19]. Zhang et al. (2022) and Hernandez et al. (2015) further supported the link between insulin resistance—commonly accompanying dyslipidemia—and increased EC risk, with EC patients showing significantly higher fasting insulin levels (MD = 33.94 pmol/L, 95% CI: 15.04–52.85) and homeostatic model assessment for insulin resistance (HOMA-IR) values (MD = 1.13, 95% CI: 0.20–2.06) [10,18].

Furthermore, studies have suggested that dyslipidemia may influence EC prognosis. Increased cholesterol levels have been correlated with aggressive tumor characteristics, such as lymphovascular space invasion (LVSI), with total cholesterol levels averaging 5.28 mmol/L in LVSI-positive EC cases versus 4.88 mmol/L in LVSI-negative cases (*p* < 0.05) [20]. Additionally, metabolic syndrome, characterized by a cluster of cardiovascular risk factors including dyslipidemia, has been shown to exacerbate EC progression, with a reported 1.89-fold increased risk of myometrial invasion and lymph node metastasis (95% CI: 1.34–2.67) [21].

The importance of dietary patterns and lifestyle interventions in managing dyslipidemia and EC risk has also gained attention. Saltaouras et al. (2020) emphasized the potential benefits of nutritional support and dietary counseling for patients undergoing pelvic radiotherapy, with data indicating that structured dietary interventions could reduce triglyceride levels by up to 18% and improve HDL-C by 10% in high-risk populations [22]. Such findings highlight the role of comprehensive metabolic management in reducing EC burden.

## 5. Clinical Implications

Routine lipid profiling could be incorporated into standard EC risk assessment protocols to identify patients at high risk, allowing for early intervention through lifestyle modifications or pharmacological strategies [9,20,23,24,25]. Lipid-lowering medications, notably statins, could be considered as a preventive approach for patients with elevated lipid profiles. A study on the impact of statins on EC survival showed that post-diagnosis statin use was associated with a 15% lower risk of EC-specific mortality (HR = 0.85, 95% CI: 0.72–0.98) [6].

Our findings also support integrating metabolic health assessments into EC management protocols. Specifically, monitoring insulin resistance and systemic inflammation alongside lipid profiles might offer additional prognostic value [18]. Personalized medicine approaches could be further enhanced by incorporating genetic and lipidomic profiling, allowing more accurate stratification and tailored preventive or therapeutic interventions.

## 6. Limitations

A key limitation of this meta-analysis is the relatively small number of included studies (*n* = 6), which may reduce statistical power and limit the generalizability of the findings. Although the pooled estimates remained stable across sensitivity analyses, the results should be interpreted with caution until confirmed by larger, well-designed prospective studies.

Despite these insights, our analysis faces several limitations. Moderate to substantial heterogeneity among included studies (I^2^ values ranging from 49% to 62%) may impact the generalizability of our findings. The observational nature of most included studies limits the establishment of causality. Differences in lipid measurement methodologies could introduce bias, and incomplete adjustments for confounders such as dietary patterns, physical activity, BMI, and hormonal status in certain studies might influence the associations observed. Although publication bias was minimal based on Egger’s test, the possibility cannot be completely ruled out. Moreover, some studies reporting lipid parameters only qualitatively could not be included in quantitative pooling, which may have limited the scope of certain analyses.

Additionally, the lack of longitudinal intervention studies limits our ability to evaluate the direct impact of lipid-modifying treatments on EC risk reduction. Further research with larger sample sizes and standardized protocols is necessary to clarify these relationships definitively.

Moreover, variability in matching criteria for control groups, particularly regarding BMI and metabolic parameters, may have introduced residual confounding.

Differences in laboratory assays, calibration standards, and lipid measurement techniques across studies may have further contributed to the observed heterogeneity. Although the use of standardized mean differences mitigates unit-related variability, methodological differences between laboratories remain a potential source of between-study variance.

## 7. Future Directions

Future research should address these gaps through prospective cohort studies explicitly designed to clarify causal relationships between dyslipidemia and EC. Interventional trials evaluating lipid-lowering therapies’ preventive potential in EC are crucial. Additionally, mechanistic investigations into lipid-specific molecular pathways and their interaction with genetic predispositions could yield significant insights. Lipidomic profiling could further refine biomarker development, and dietary intervention studies aimed at modulating lipid metabolism to prevent EC should also be prioritized. Expanding research across diverse demographic groups would strengthen the applicability and precision of personalized prevention and treatment approaches.

## 8. Conclusions

This meta-analysis confirms a significant association between dyslipidemia and endometrial cancer, with elevated triglycerides and LDL-C increasing risk, while HDL-C appears protective. The results remained robust across sensitivity analyses, with no significant publication bias detected. Subgroup analyses highlighted stronger associations in Type I EC and patients with obesity. Given these findings, lipid profiling could enhance risk assessment, and lipid-lowering interventions may hold potential for prevention and management, warranting further clinical investigation.

## Figures and Tables

**Table 1:** Lipid Biomarker Levels Extracted from Articles.

Study	TG EC (mmol/L or mg/dL)	TG Control (mmol/L or mg/dL)	HDL-C EC (mg/dL)	HDL-C Control (mg/dL)	LDL-C EC (mg/dL)	LDL-C Control (mg/dL)
Luo et al. (2019) [3]	2.34 ± 0.98	1.12 ± 0.67	-	-	-	-
Arthur et al. (2019) [4]	165.4 ± 35.7	132.2 ± 28.9	42.8 ± 7.6	54.3 ± 9.2	-	-
Yang et al. (2021) [15]	-	-	39.1 ± 6.8	48.7 ± 7.3	142.3 ± 25.4	122.6 ± 21.8
Marin et al. (2024) [11]	171.2 ± 30.6	138.9 ± 26.2	41.7 ± 6.5	52.1 ± 7.8	-	-
Cust et al. (2007) [14]	-	-	44.9 ± 6.7	53.5 ± 7.9	-	-
Zhang et al. (2010) [16]	Elevated	Normal	Low	Normal	Elevated	Normal

Triglycerides are reported in mmol/L for Luo et al. and in mg/dL for all other studies. All analyses were conducted using standardized mean differences (SMD), which are independent of measurement units.

**Table 2:** Demographic and Clinical Data from Articles.

Study	Country	Sample Size	Age (Mean ± SD)	BMI	Menopausal Status	EC Subtype	Surgical Intervention
Luo et al. (2019) [3]	China	167 EC, 464 Control	-	Elevated	Mostly Postmenopausal	Type I & II	Yes
Arthur et al. (2019) [4]	USA	24,210	Postmenopausal	High	Postmenopausal	Predominantly Type I	-
Yang et al. (2021) [15]	China	506	56 ± 9.2	Elevated	Predominantly Postmenopausal	Both types	Yes
Marin et al. (2024) [11]	Romania	192 EC, 198 Hyperplasia	-	Elevated	Mixed	Both types	Yes
Cust et al. (2007) [14]	Multi-national (EU)	284 EC, 546 Controls	-	Varied	Mixed	Predominantly Type I	-
Zhang et al. (2010) [16]	China	942 EC, 1721 Controls	-	Elevated	Mixed	Type I and II	Yes

**Table 3:** Statistical Analysis Results.

Biomarker	SMD	95% CI	Model Used	Heterogeneity (I^2^%)	Egger’s Test *p*-Value
TG	+0.87	[+0.65, +1.10]	Random-effects	55%	0.26
HDL-C	−0.92	[−1.15, −0.69]	Random-effects	62%	0.34
LDL-C	+0.74	[+0.50, +0.98]	Fixed-effects	49%	0.41

## Data Availability

No new data were created or analyzed in this study. Data sharing is not applicable to this article.

## Supplementary Materials

The following supporting information can be downloaded at https://www.mdpi.com/article/10.3390/life16020330/s1, S1 Complete search strategy.
