# Supplementary material for: Fats and Facts: A Meta-Analysis of Lipid Biomarkers in Endometrial Cancer
Source: Life (Basel). 2026 Feb 14;16(2):330. doi: 10.3390/life16020330 (PMC12942389; doi:10.3390/life16020330)
Supplement: Supplementary file 1 [file life-16-00330-s001.zip › life-4142197-supplementary.pdf]

Pubmed:

("Endometrial Neoplasms"[MeSH] OR "endometrial cancer" OR "endometrial carcinoma")
AND
("Lipids"[MeSH] OR "Dyslipidemias"[MeSH] OR triglycerides OR "HDL cholesterol" OR "LDL cholesterol" OR cholesterol)
AND
("Biomarkers"[MeSH] OR "lipid biomarkers")
Filters: Humans, English language

EMBASE:

('endometrial cancer'/exp OR 'endometrial carcinoma' OR 'endometrial neoplasm')
AND
('lipid'/exp OR 'dyslipidemia'/exp OR triglyceride OR hdl OR ldl)
AND
('biomarker'/exp)
AND
[humans]/lim AND [english]/lim

Scopus / WoS:

TITLE-ABS-KEY (("endometrial cancer" OR "endometrial carcinoma")
AND
(triglycerides OR cholesterol OR HDL OR LDL OR dyslipidemia)
AND
(biomarker*))

Cochrane Library:

(endometrial cancer OR endometrial carcinoma)
AND
(triglycerides OR cholesterol OR HDL OR LDL OR dyslipidemia)
